# A global network model of abiotic phosphorus cycling on Earth through time

**DOI:** 10.1038/s41598-022-12994-9

**Published:** 2022-06-07

**Authors:** Marcos Jusino-Maldonado, Rafael Rianço-Silva, Javed Akhter Mondal, Matthew Pasek, Matthieu Laneuville, H. James Cleaves

**Affiliations:** 1 Planetary Habitability Laboratory, University of Puerto Rico at Arecibo, Arecibo, Puerto Rico; 2 Blue Marble Space Institute of Science, Seattle, USA; 3 Departamento de Física, Faculdade de Ciências, Universidade de Lisboa, 1749-016 Lisbon, Portugal; 4 Department of Geology, University of Calcutta, Kolkata, 700019 India; 5 University of South Florida, Tampa, USA; 6 Earth-Life Science Institute, Tokyo Institute of Technology, Tokyo, Japan; 7 Earth and Planets Laboratory, Carnegie Institution of Washington, Washington, DC USA

**Keywords:** Biochemistry, Biogeochemistry, Element cycles, Astrobiology, Planetary science, Exoplanets

## Abstract

Phosphorus (P) is a crucial structural component of living systems and central to modern bioenergetics. P cycles through terrestrial geochemical reservoirs via complex physical and chemical processes. Terrestrial life has altered these fluxes between reservoirs as it evolved, which is why it is of interest to explore planetary P flux evolution in the absence of biology. This is especially true, since environmental P availability affects life’s ability to alter other geochemical cycles, which could then be an example of niche construction. Understanding how P reservoir transport affects environmental P availability helps parameterize how the evolution of P reservoirs influenced the emergence of life on Earth, and potentially other planetary bodies. Geochemical P fluxes likely change as planets evolve, and element cycling models that take those changes into account can provide insights on how P fluxes evolve abiotically. There is considerable uncertainty in many aspects of modern and historical global P cycling, including Earth’s initial P endowment and distribution after core formation and how terrestrial P interactions between reservoirs and fluxes and their rates have evolved over time. We present here a dynamical box model for Earth’s abiological P reservoir and flux evolution. This model suggests that in the absence of biology, long term planetary geochemical cycling on planets similar to Earth with respect to geodynamism tends to bring P to surface reservoirs, and biology, including human civilization, tends to move P to subductable marine reservoirs.

## Introduction

Phosphorus (P, mainly in a + 5 phosphate oxidation state) is a key component of modern metabolism^1,2^ and is among the main limiting factors for marine and terrestrial primary productivity in surface environments^3,4^. P may be an ideal building block for biochemistry^5^, is an essential component of the backbone of all known genetic material (e.g. DNA and RNA), and contributes to the strength of bones and teeth in vertebrates, which helped them adapt to life on land^6^. Thus, P has likely played a role in multiple major transitions in the emergence of life on Earth. Since P availability is limiting for life in the modern oceans, P availability has been appealed to as a driver of evolutionary radiation^7^ and possibly a limiting factor for the origins of life (see^8^). It is also possible the first organisms did not use P at all^9^, but the coupling of biology with P cycling represented a major linkage of life with geochemistry. Whether the P content of a planet or moon is ever a bottleneck for the emergence of life may hinge on P’s abiological chemodynamics.

P, similarly to nitrogen (N), is a pnictogenic element due to its outer shell electron configuration, and this strongly affects its chemodynamic behavior. In contrast with N, the highest oxidation state oxides of P (+ 5) are often extremely insoluble in combination with alkaline earth metal ions (especially Mg^2+^ and Ca^2+^, which are the most abundant cosmically and terrestrially), as well as when combined with trivalent ions such as Fe^3+^ and Al^3+^. In contrast with N, the behavior of inorganic P species in abiotic environments with respect to how distinct reservoirs interact with each other and how the distributions of these change through time have been only poorly explored, which motivated this study.

P is thought to be formed by neutron capture by ^29^Si and ^30^Si in massive stars, and thus its relative planetary abundance to other elements is initially set by presolar processes^10^. However, its later behavior in planetary contexts depends on partitioning behavior during differentiation (see^8^). In some contexts P likely behaves as a volatile element during planetary accretion. Under some melt conditions, it is an incompatible element, and sometimes it also behaves as a siderophile element^11^. This latter behavior may best explain its depletion in Earth’s upper geological reservoirs (e.g. the bulk silicate Earth, BSE, which is dominated by the mantle) relative to the materials from which Earth is thought to have formed. Ultimately a planet’s P abundance may be connected to pre-solar metallicity distributions, e.g. the so-called Galactic Habitable Zone^12^.

First principles assessment of the terrestrial P inventory starting from CI chondritic P abundances typically assumes the terrestrial P content is estimable by scaling CI meteorite inventories to Earth’s mass. McDonough and Sun^13^ estimated a bulk P content of 1,080 ppm for CI material, which scaled to Earth’s mass (~ 5.97 × 10^24^ kg) would give an initial terrestrial P inventory of ~ 6.45 × 10^21^ kg P^13^. Stewart and Schmidt^14^ suggest that if the core formed rapidly, and little P was lost to ablation, ~ 90% of Earth’s initial P is sequestered in the core (~ 5.8 × 10^21^ kg P, accounting for ~ 0.3% of the core’s mass, which is compatible with experimental measurements). If there has been little material exchange between the core and mantle since core formation (an assumption we make here), the chemodynamics of Earth’s remaining initial residual BSE P content (~ 6.5 × 10^20^ kg P) is the question at hand, though we also explore deviations from a 90% partitioning value below.

Although multiple studies have explored the P composition of deep Earth reservoirs (e.g.,^8,15,16^, interior planetary reservoirs (such as the core, the lower mantle (LM), and upper mantle (UM)) and the exchange of materials between them are not presently well understood^17,18^. There is also some debate over initial and current P inventories (see^8^). The current model explores the influence of these uncertainties on the abundance of P in terrestrial surface reservoirs.

The oxidation state of P in the environment is governed by the kinetics and stoichiometry of electrons from other easily exchanged donor and acceptor elements, most importantly Fe, S and C^19^. Consideration of the oxidation state of the BSE over time suggests that P remaining in the BSE after core formation would have continuously experienced global redox conditions maintaining it in the + 5 oxidation state^8,11^, though localized reservoirs hosting reduced P are also possible. In modern terrestrial surface environments, P is generally encountered in the + 5 state, though there is some evidence for widely distributed, though minor, reduced P^20^, and the widespread distribution of genes for the oxidation of reduced phosphorus suggests there is a significant biologically exploitable reservoir of reduced P^21^. However, such reduced P reservoirs are either very small, quickly turned over, or have been systematically overlooked by geochemists for some time.

If P is required for the emergence of life, the supply of P to aqueous surface reservoirs is likely set by geodynamic processes, and especially by phosphate solubility in various reservoirs, especially melts and the oceans. The distribution of P in surface reservoirs is also governed by erosional processes, and now biology, which has become connected to planetary P processes, though direct biological influence on the P cycle is likely limited by the resupply of P to habitable planetary reservoirs (e.g. those presenting pressure and temperature conditions allowing for the existence of life). This study aims to describe how P becomes distributed on rocky planets with active geochemical cycling, based on a previous box model constructed by Laneuville et al*.*^22^ for abiotic nitrogen cycling on terrestrial planets. Modeling P fluxes in this way may also help understand the potential differences between “geosignatures'' and “biosignatures”^23,24^. Such flux models are necessarily crude since much remains unknown about the interactions and contents of various reservoirs, but may highlight the most important gaps in the present knowledge of P chemodynamics.

This model thus does not consider localized unusual concentration mechanisms, e.g.^25^, but rather the overall flux of P between reservoirs. Phosphate often forms incompatible phases with melts, leading to P-concentration in pegmatites^8^, though it is not clear that P is generally concentrated in material volcanically returned to Earth’s surface. Though P may become concentrated in some rock types, it is normally distributed in others, and overall the flux may be globally representative of a mixed mantle source as a whole. Likewise, as an example of why such smoothing is warranted in this context, a melange of continental crust (CC) of differing composition presently contributes P to the oceans^26^ via weathering in a combined fashion. Since this model does not distinguish between types of weatherable crust, weathering is modeled as an average across combined lithologies.

The model presented here can be used to model any rocky planetary body for which reservoir dynamics can be estimated, as well as for any chemical species with a single geochemically common oxidation state, and thus may be useful for understanding planetary chemodynamics on other Solar System planets and moons, and exoplanets.

## Results and discussion

This study’s aim was to model the geochemical cycling of P on rocky planets with similar geodynamical activity as Earth, but in the absence of life (by neglecting biogeochemical processes over Earth’s evolution). This model explores the period beginning after the Moon-forming impact (~ 4.5 Ga^27^), and does not consider nuances of the potential LHB^28^ or late veneer models (e.g.^29^) suggested to have contributed small but significant amounts of undifferentiated material between 4.1 and 3.8 Ga. The examples presented here further assume an onset of modern-style plate tectonics early in Earth history (at t = 4.5 Ga, the time of Earth’s formation), though this is a contentious issue^30^, and a modifiable parameter of the model. Parameter sensitivity analysis examining the final state of P reservoirs after 4.5 Ga and distinct planetary reservoirs’ P distribution was also examined (see Methods).

**Figure 1:**
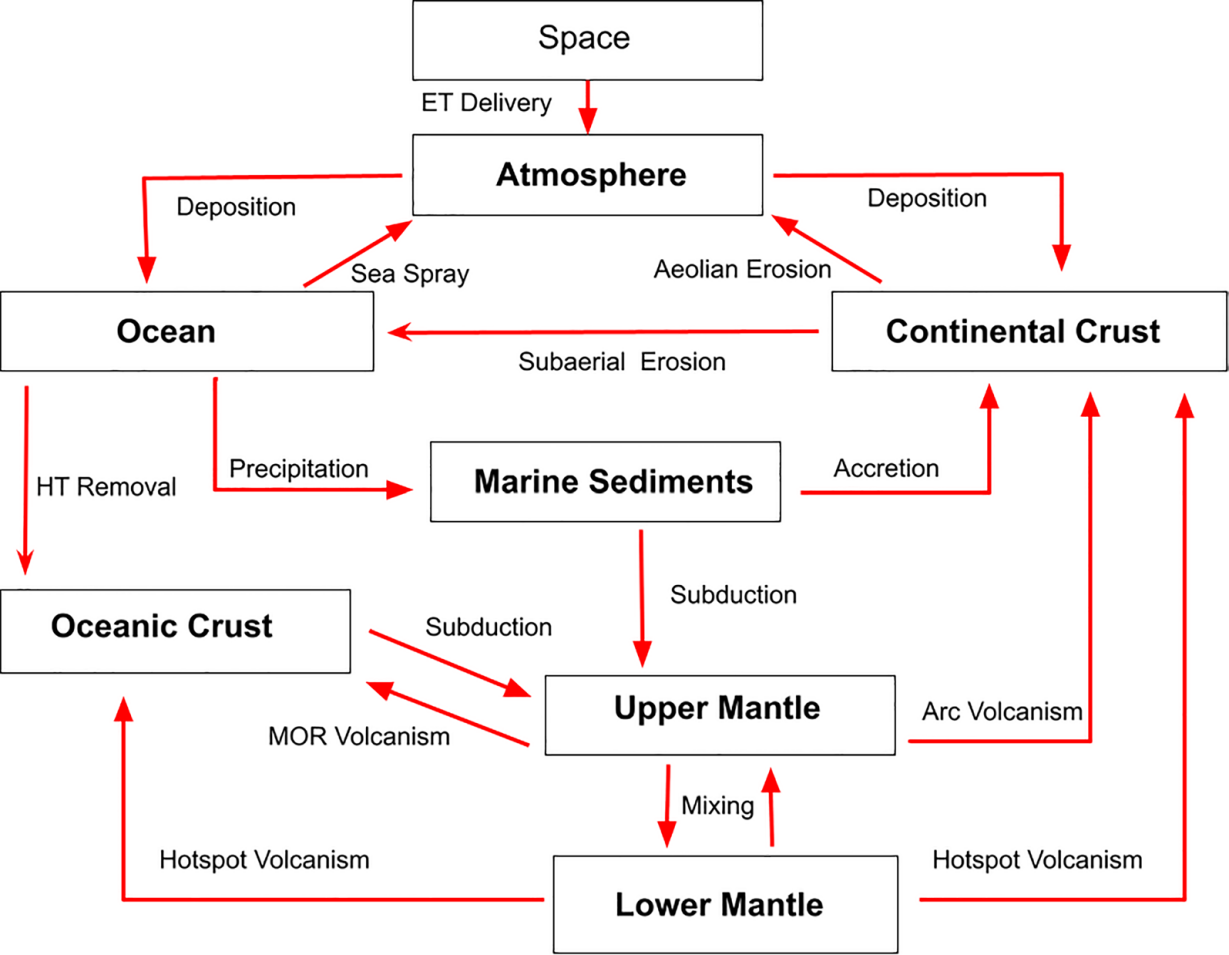
Reservoirs (boxes) and fluxes (arrows) considered in this global box model. The reservoirs are abbreviated in the text as follows: continental crust (CC), marine sediments (MS), oceanic crust (OC), upper mantle (UM), lower mantle (LM).

Figure 1 shows how reservoirs are coupled through different processes in this model. Although there are many locally interacting geochemical processes, their global significance depends on both fluxes and reservoir sizes. The parameterization of these reservoirs and fluxes is presented below.

**Figure 2:**
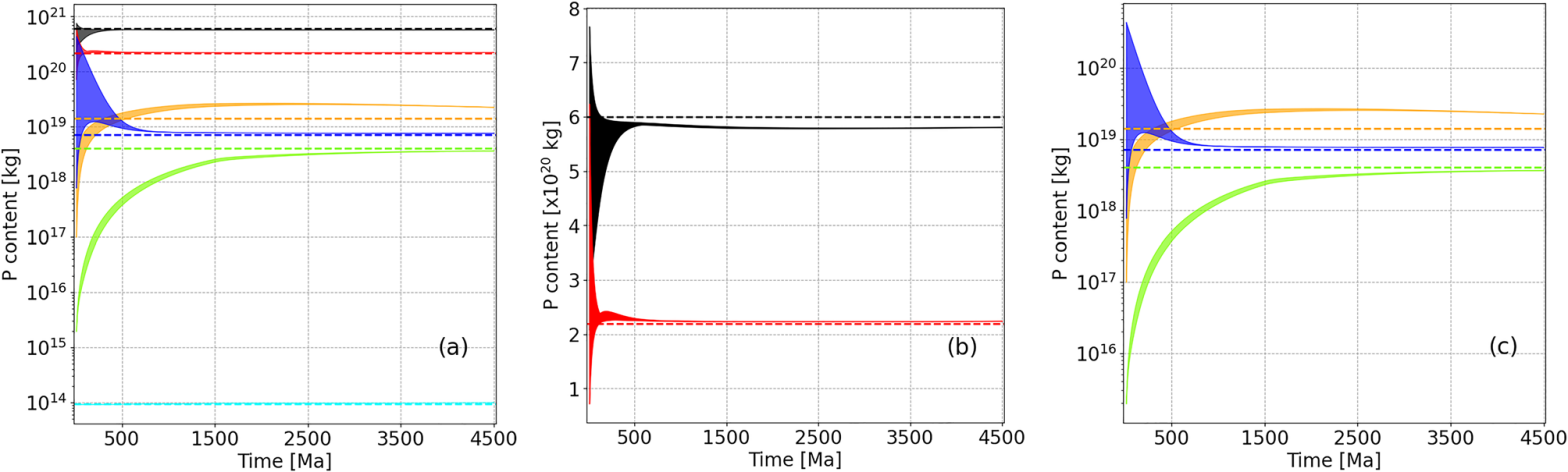
Evolution of P reservoirs in the main model assuming an initial 10% residual chondritic P inventory randomly seeded across BSE reservoirs across 50 randomly seeded model runs. (**a**) All reservoirs (Black: LM, Red: UM, Orange: CC, Blue: OC, Green: MS, and Turquoise: Oceans). (**b**) Expanded view of UM and LM reservoir evolution. (**c**) Expanded view of CC, MS and OC reservoirs. Dotted lines represent estimated modern values (see Table 1 in Methods). The atmospheric reservoir is not shown since it is very small, and saturates quickly then remains constant.

We first present the time evolution of our model’s reservoirs P content with “best guess” model parameters and how P distribution over the distinct reservoirs compares to that of the present-day, biodiverse Earth. Having defined and parameterized modern and initial reservoir inventories, and the ways fluxes between reservoirs are potentially interconnected and may have changed over time, we examined the ways reservoir P contents evolve (see Methods). The basic model using parameters governing the evolution of fluxes based on literature estimates (see Tables 1, 2), and their variation based on randomized initial reservoir seeding is shown in Fig. 2.

All randomly seeded initial configurations eventually converge on apparent steady states. Mantle reservoirs converge to apparent steady states in ~ 100–500 Ma (which reflects the mixing time scale), while the smaller and more dynamic upper reservoirs converge over 1–2.5 Ga (which likely reflects the need for the much larger mantle reservoirs to reach steady-state first). The modeled mantle steady states vary from the modern estimates in Table 1 by -3% (LM) and + 2% (UM), which are relatively small deviations, but which strongly affect surface reservoirs. Upper BSE reservoirs are overestimated by this model (MS − 10%, CC + 42%, OC + 5.6%), but as the mantle P mass is at least an order of magnitude higher than the combined crustal P mass on modern Earth, small discrepancies in fluxes can easily account for these offsets. As the ocean concentration parameter is fixed in the model by the estimated solubility of P, a fixed concentration is continuously obtained as the oceans always reach saturation. It should be noted that these curves display the total P mass in each reservoir, not the mass of the reservoir itself.

Models assuming 85%, 95% and 97% initial chondritic P inventories are sequestered in the core are presented in Figures SI1–SI3. As discussed below, the 90% residual BSE P model already suggests significant biological impact on P cycling, and greater core-P sequestration models imply an even larger biological influence on global P cycling, or that many literature values for flux evolution or reservoir P content are presently poorly estimated.

The oceans and the atmosphere are, as on present-day Earth, small and transient, but highly connected reservoirs of P with maximum concentrations predetermined by physical parameters in the model. This causes these reservoirs to work as “switchboards” through which other reservoirs exchange P dynamically and rapidly.

There are characteristic timescales of ~ 100–500 Ma and ~ 2.5 Ga which the modeled systems take to converge to apparent steady states, but there is also a long term evolution of those steady states dictated by the flux parameterizations. This is most visible in the continuous change of CC P content, as its replenishment sources (volcanism and accretion) change over geological time, unlike the CC’s main P removal mechanism, erosion, which is mainly dependent on CC mass and surface area. Erosional rates thus increase with emerged surface area replenishing sedimentary basins with P. This eventually causes CC P content to decrease slightly after ~ 1.5 Ga and converge around a common value. This decrease does not appear to affect the OC in the same way, due to its faster cycling driven by seafloor spreading and destruction.

### Parameter sensitivity

Having built a “main” baseline model and randomly seeded it, it is apparent that over long time periods the model tends towards apparent steady states which both under- (in the case of the LM and MS) and overshoot (for the UM, OC, and CC) estimated modern terrestrial P reservoir content. Though it was the point of this study to examine planetary P cycling in the absence of biology, it is worth examining how uncertainties in the model’s parameters affect its evolution before considering how biological P cycling might affect the outcome. We explore this here and graphically document it in Figures SI4–7 (see also Figures SI3–15).

Varying some fluxes strongly affects some reservoirs, while others are relatively resilient to change. Naturally, as the parameterized solubility of P is varied, the size of the ocean P reservoir changes accordingly. However, as the ocean reservoir is ~ 10^4^ to 10^6^ times smaller than any of the solid Earth reservoirs, even varying the maximal concentration over four orders of magnitude has only a < 10% impact on the MS and OC P reservoir content, which it most strongly affects (see Figure SI4).

As might be expected, due to their large sizes relative to surface reservoirs (see Table 1), the final converged values of the UM reservoir only change by a factor of ~ 10% or less even when varying the erosion rate over four orders of magnitude (Figure SI5a), the CC accretion efficiency from 0 to 100% (Figure SI6a), or the mean subduction time from 50 to 150 Ma (Figure SI5d). Varying the mantle mixing rate over three orders of magnitude causes a significant difference in the LM and UM P content (Figures SI7a,b), though smaller, more reasonable variations cause only ~ 10% changes. Varying the initial volcanism rate or the volcanism P enrichment factor over more than an order of magnitude causes a similarly small difference in the P content of the UM (Figures SI7g,SI14a). Estimates of the P content of the UM (which are the most measurable) are thus a strong constraint on this modeling.

OC P content is strongly affected by the OC subduction rate (Figure SI5e), and to a much lesser extent by the CC erosion rate (Figure SI5h), varying by a factor of three with respect to the former and a few percent with respect to the latter, mainly due to the mantle P enrichment that occurs at higher erosion rates. Variability in initial volcanism rates does not affect final OC P levels, but they are strongly affected by the volcanic enrichment factor (SI14b), increasing by ~ an order of magnitude as this factor increases from 1 to 10x.

CC P content is strongly affected by CC erosion rates and accretional efficiency (Figures SI5B,6c), varying by more than a factor of approximately 6–8 in each parameterization.

The P content of MS is strongly affected by CC erosional flux, OC subduction rate and CC accretion efficiency, and weakly affected by the mantle mixing rate and maximum oceanic P solubility (see Figures SI5f.,7e). Factors which lead to high MS P content generally also coincide with factors which lead to higher CC P content, highlighting the point that these two reservoirs are major determinants of each other.

Finally, assuming very low erosion rate predictably enhances the P content of the CC at the expense of MS (Figure SI10), and depletes mantle reservoirs significantly (Figure SI10a).

Despite the many uncertainties surrounding initial inventories of P reservoirs on early Earth, or the fact that many geophysical processes such as magmatism or plate tectonics are poorly constrained over deep time, the random initial seeding and parameter sensitivity analysis used here allows the model to explore many distinct configurations, and provides a framework to explore the effects of the absence of life on planetary P geochemical cycling.

There are three major variables in this model: reservoir size, reservoir flux and reservoir connectivity, all of which may have changed markedly over time. Randomly assigning a large set of distinct initial reservoir masses over multiple simulations enhances the probability some aspects of this model overlap with the real trajectories of P reservoir distributions over time. This consideration is also true for the geophysical parameters considered in this model as many of these were varied to explore parameter sensitivity here. Notably, early variation in time-dependent values is observed (Fig. 2), even in the most extremely seeded cases. These values often replicate present terrestrial P reservoir levels, which suggests biology strongly affects some BSE reservoirs, and only very weakly affects others.

Having explored various model parameters, we can finally ask the question of how biology needs to impact global cycling in order to reconcile this model with observations. The simplest explanation is that biology allows for tighter coupling between riverine and aeolian erosional runoff and CC accretion via P enrichment in MS, which may slow or speed P loss to interior planetary reservoirs (e.g. UM and LM), and also increases the erosional flux of P, which is shunted to continental accretion and arc volcanism, as well as subduction. The retention of P in upper planetary reservoirs must be disfavored in various non-negligible ways to bring this model’s results into register with modern observed P reservoir values.

A novel example of biological flux of P from oceans to land in the form of guano originated with the advent of flying reptiles and later birds. Modern fluxes via this route are estimated to be on the order^31^ of 10^8^ kg P year^−1^. Rough estimation of the total flux by this route over the ~ 200 million years over which flying reptiles and birds have existed (assuming the ecosystem-wide P fluxes from oceans to CC are comparable to modern ones continuously over that time period) would give a cumulative value of ~ 2 × 10^16^ kg P transported from the oceans to CC. This is ~ 0.1% of the modern CC inventory, and thus small but non-negligible, and coincidentally comparable to the putative LHB P flux over a similar timescale. P fluxes in this direction from other biological (for example by spawning fish, e.g. salmon) are likely much smaller and generally deposited in riverine environments where the reverse flux is likely quite rapid.

We note that recent work by Hao et al.^32^ examined the geochemistry of marine P in the Archean and its potential limitation of net primary productivity (NPP) during that period. While the inorganic P concentrations and solubilities are estimated in that work using somewhat more involved calculations, the final estimated values lie quite close to those estimated and used here (compare Figure SI11 here with Fig. 1 in^32^). Their work also comes to the same conclusion that there was likely little P transport from the OC to the oceans. We note too that the low NPP value estimated in that work implies biology largely did not alter the global P cycle significantly during Earth’s earliest history.

The inventory of heavy elements (in astronomical terms, generally those heavier than helium, thus including P), a planet is endowed with depends on a concatenated series of events, including heavy element generation in the interiors of stars of sufficient size, their broadcast via supernova events, and the accretion of those broadcast chemical elements into nascent planets. Heavy element abundance is unevenly radially distributed across galactic disks due to the relative ages and density of stars in such disks, and inasmuch as rocky planets are dominated by heavy elements, the initial P endowment of a planet is related to the concept of the Galactic Habitable Zone (GHZ)^12^.

It is not yet clear where Earth lies in the continuum of planets endowed with biogenic elements such as P. Earth may be an average, low or especially well-P-endowed planet. P is neither a common nor an uncommon element in terrestrial rocks, but P is highly enriched in biological material, and biology has evidently developed mechanisms via natural selection to extract and concentrate rare elements (including P) from the environment, giving biological material a markedly different elemental profile from bulk seawater (e.g.^33^). Thus a planet endowed with more or less P than Earth may be equally likely to develop and allow for the complexification of life, if biology evolves to efficiently extract P from its surroundings. Once biology has evolved methods to efficiently extract rare elements from the environment, oversupply of that element can certainly perturb ecosystems. It may thus be difficult to argue that metal-rich or metal-poor GHZ environments bias how common habitable planets may be with respect to their initial endowment with P. Biological P cycling inherently involves organisms being able to extract P efficiently from P-poor environments. This suggests that the low erosional rates and P concentrations expected to occur in the subsurface oceans of icy moons in our solar system should not be excluded as possible habitats for life solely due to the likely paucity of dissolved P^34^.

On short time scales, it is thus reasonable to expect that environmental fluctuations in the availability of P may impact the development of planetary ecosystems, as they apparently may have done on Earth, but over longer timescales, biology may be able to adapt to low-P environments.

## Conclusions

Models like this are necessarily constrained by the accuracy of observable data and estimates of reservoir sizes and fluxes over time, which are subject to debate based on incomplete data. Nevertheless, such models provide a simple way to explore how geochemical P cycling proceeds in terrestrial planetary environments, such as those of Venus and Mars, and exoplanetary settings. Because of differences in accretional history, planetary mass, and tectonic history, different terrestrial planets might be expected to have different initial P reservoir inventories, and thus different P content in bioavailable surface environments over time (see for example^35,36^). Similar chemodynamic considerations should apply to the behavior of P in other terrestrial planetary environments. A great deal likely remains to be understood regarding the deep planetary interior behavior of P in rocky planets of varying composition and mass.

Changes in the availability and exploitability of biologically available P have been implicated in major transitions in biological complexity, including the origins of life and the Cambrian explosion^37^. The impact of biology on global surface P transport likely cannot be overestimated. Undoubtedly early marine microbial organisms led to a greater sequestration of P in upper planetary reservoirs as organisms both extracted P from minerals and deposited them in MS, removing P from the monotonous cycle of deep Earth delivery and subduction. It is thus potentially overreaching to suggest that extraterrestrial ecosystems would not be equally able to evolve among their own peculiar P chemodynamics if P is ubiquitously a bioessential element.

The most significant impact biology has had on P cycling is likely the increase in the rate of CC weathering. There is evidence for an extensive terrestrial microbial biome stretching back ~ 1.2 Ga^38^, and scarcer evidence back to ~ 2.6 Ga^39^, which likely had a significant impact on CC weathering, even before the advent of the first embryophyte land plants ~ 0.7–0.46 Ga^40^, whose flourishing undoubtedly impacted CC weathering. This bracketed period is coincident with the peak CC P content predicted by the main model here (Fig. 2).

Human agricultural practices have increased erosional CC P flux by estimated factors^41^ of 10–100. Modern agriculture is heavily dependent on P-fertilization, and P has been used to augment agricultural yields since the beginnings of civilization^42^. Many agriculturally-relevant and geologically recent P-rich sources are dependent on bioaccumulation provided by coastal food chains^31^, in which oceanic upwelling provides nutrients from MS which become bioaccumulated then deposited in CC regions by coastal avians (in the form of guano^31,43^) and decaying matter from dead marine organisms.

Concerns have been raised over the depletion of easily exploitable P reservoirs for this reason^44^. It is clear that these easily exploitable surficial P reservoirs will deplete faster due to human activity than they can be naturally replenished, which may become a problem for future civilizational food production. Biogeochemical P cycling may be an example of niche construction^45^ and in a planetary context, a mechanism by which biology modifies its environment to make its environment more favorable for the propagation of biology itself.

We are currently expanding this model to examine the potential impacts of P redox state changes as well as how this model can be expanded to consider P fluxes in exoplanetary and icy moon environments.

**Table 1:** Estimated P content of considered modern terrestrial reservoirs.

Reservoir	Estimated modern reservoir P content (kg P)	Dominant P oxidation state	Modern reservoir mass (kg)
Core	5.8 × 10^21^ ^13^	0	2.0 × 10^24^ ^46^
Lower mantle	6.0 × 10^20^ ^16^^(1)^	+ 5	3.0 × 10^24^ ^47^^(2)^
Upper mantle	2.2 × 10^20^ ^16^^(1)^	+ 5	1.1 × 10^24^ ^48^
Oceanic crust	7.2 × 10^18^ ^9^^(5)^	+ 5	9.0 × 10^21^ ^50^
Marine sediments	4.0 × 10^18^ ^51^	+ 5	2.6 × 10^20^ ^52^
Continental crust	1.2–1.4 × 10^19^ ^53^^(3)^ 1.6 × 10^19^ kg ^54^	+ 5	1.9 × 10^22^ ^55^–2.2 × 10^22^ ^45^^(4)^ 1.6 × 10^22^ ^54^
Atmosphere	2.8 × 10^7^ ^56^	+ 5	5.1 × 10^18^ ^57^
Oceans	9.3 × 10^13^ ^58^	+ 5	1.4 × 10^21^ ^57^
Total	6.6 × 10^21^ kg of Total P on Earth, 8.4 × 10^20^ kg starting BSE P		

Values are rounded to two significant figures. ^(1)^Calculated assuming the 200 ppm UM P value cited in^16^. ^(2)^Calculated from the mass of the mantle given in Lodders^47^ minus the value estimated in^48^. ^(3)^Computed using the range of CC masses cited and using the average 0.15 wt % P_2_O_5_ value in^53^. ^(4)^The total mass of Earth's crust (oceanic + continental) is estimated as 2.8 × 10^22^ kg^50^. Using crustal thickness to define CC, the mass of CC is 2.2 × 10^22^ kg if the minimum thickness is 12–18 km, 2.1 × 10^22^ kg for 22.5 km, 2.0 for 25 km, and 1.9 × 10^22^ kg for 30 km. These numbers include all sediments as continental crust. Using the C2 definitions in^50^ to distinguish OC and CC (and including oceanic plateaus which contain some CC), we calculate the CC mass as 1.9–2.2 × 10^22^ kg. Using 2.8 × 10^22^ kg as the value for the total mass of the crust and subtracting 1.9 × 10^22^ kg as the mass of the CC leaves ~ 9 × 10^21^ kg as the total mass of the OC, and, using the averaged 0.18% wt of P_2_O_5_ in OC from^49^.

**Table 2:** Main simulation parameters and their respective estimated parameterizations.

Parameter	Estimated value
Surface erosion rate, **α**	2.2 × 10^−4^ m year^-1^
Wind erosion rate, **β**	2.7 × 10^−5^ m year^-1^
Atmospheric saturation P mass	2.8 × 10^7^ kg
Oceanic saturation P concentration	2.2 µM
Mantle mixing rate, Min value **F**_**0**_ Max value **F**_**1**_	1.0 × 10^−8^ year^−1^ 3.0 × 10^−8^ year^−1^
Subduction rate, **D**	100 Ma
Continental accretion efficiency, ε	0.30
Initial volcanic multiplying factor, **f**	3 ×
Volcanism P enrichment factors: MOR volcanism Arc volcanism Hotspot volcanism	4.6 × 5 × 4.6 ×

## Methods

**Figure 3:**
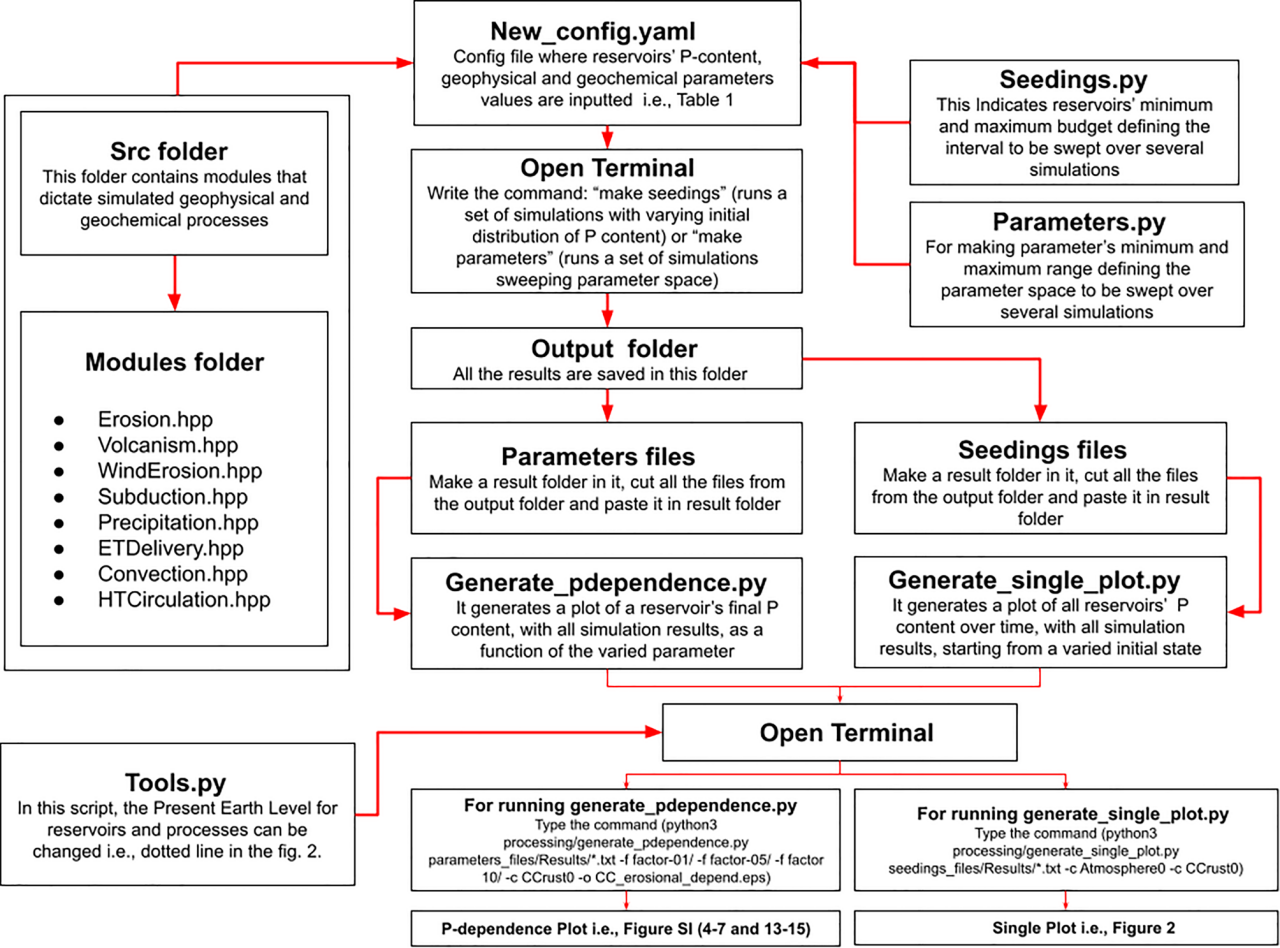
Flowchart outlining the computational workflow used in this model. Further details can be found in^22^ and in the Supplementary Information.

This model requires the definition of considered reservoirs and the fluxes between them. We start by reporting various sources for reservoir definitions and continue with consideration of the list of processes governing P-fluxes between reservoirs. Details of the mathematical model are also provided in links to the open source code and further technical details are provided in the Supporting Information. A brief overview of the computational flow is shown below in Fig. 3.

Table 1 shows the masses of modern Earth system reservoirs and their P contents estimated in previous analyses which were used to parameterize this study. Distinct boundaries between these reservoirs can be debated. It is also part of the goal of this study to reconcile such debates, but there are obvious distinctions between many of them.

The data in Table 1 are based on previous studies on late Hadean and Archean materials (e.g.^59,60^), or estimated using modern values, which have already been affected by biology (e.g.^61^). Early Earth P reservoir content is hard to constrain since the original reservoirs have been reworked by Earth’s dynamic evolution, and various interdependent geochemical fluxes may have varied over time.

Early Earth P reservoir content estimation is likely more reliable for more accessible surface reservoirs compared to deep Earth reservoirs. A major uncertainty is the estimated sizes of surface reservoirs and their composition and growth over time, which may have also changed over time in ways this model does not accurately model. For example, the mass growth of CC over time is not well constrained (e.g.^62^). Based on Armstrong et al.^63^ our main model parameterizes CC growth over time as a piecewise-defined function, starting from an arbitrarily small non zero fraction of emergent land, growing linearly to 2/3 of its present-day value over 1.5 Ga then continuing to grow linearly but at a slower pace towards the present-day ~ 40% emergent CC area. An alternative scenario in which CC growth rate decays exponentially according to the model of Campbell^64^ converging to present values is also presented in Figure SI12. Notably, these two models, though markedly different, produce very similar bulk reservoir P distributions.

Crustal volumes can be estimated from Earth’s surface area covered by the material of a differing density multiplied by thickness. First-order considerations only examine Earth’s total surface area (~ 5.1 × 10^8^ km^2^) divided into the subaerially exposed land area (~ 29%, or ~ 1.48 × 10^8^ km^2^) as CC, and consider the remaining crust (~ 71%, or ~ 3.6 × 10^8^ km^2^) as OC. However, a significant portion of CC is presently, and intermittently, submarine due to ice ages and concomitant sea-level changes, e.g. continental shelves and associated MS occupy ~ 2.7–5.2 × 10^7^ km^2^ of Earth’s surface, according to^58^, which would under-apportion the surface area of CC from the subaerial emergent land area by 18% to 35%.

However, submerged CC is subject to lower erosional flux, and thus this difference may cancel out or not need to be factored into CC to ocean flux values. Importantly, CC shelves accumulate a significant fraction of P from subaerial CC erosion via precipitation of particulates delivered by riverine fluxes, which are typically shallow water regions that may become subaerial during ice ages, and thus subject to more rapid subaerial erosion^65^. Thus there is significant coupling between continental margin P deposition from CC erosion and P accumulation to CC during continental accretion from MS. This model does not consider periodic sea-level rise and fall, but this consideration could be easily modeled using this code.

Deep Earth composition models rely on direct sampling of xenoliths, seismological data, and cosmochemical constraints; the relative importance of each source of information varies with the depth of the considered reservoir (e.g.^66^). Consideration of the UM and LM as distinct reservoirs hinges on two observations: observed seismic discontinuities (e.g.^67^) and the differing P contents of rocks apparently derived from mantle sources over time. Presently, there is little evidence that the average P content of the CC, OC, UM, or LM (assuming these are unique identifiable reservoirs) has varied measurably across geological time compared to measured modern values^55^, which provides important model constraints.

The P content of ancient crustal materials may have decreased over time due to weathering and alteration of initially high P surface emplacement^68^. There may also have been a several-fold secular increase in the P content of subaerially emplaced igneous rocks over time due to secular cooling of the mantle and its effects on P solubility in melts^69^.

Uncertainties in the P content of large mantle reservoirs can easily absorb uncertainties in fluxes between smaller reservoirs (e.g. atmosphere, oceans, OC, CC). Given uncertainties in initial mantle P concentration values and the evolution of mantle mixing, tracking UM and LM P reservoir sizes over time is fraught.

Oceanic and atmospheric reservoirs of P and their respective disproportionating processes have likely been relatively invariant over Earth history^61,70^ with a potentially positive excursion in the Neoproterozoic^71^, largely due to the solubility limit of apatite, which is variable with respect to pH, temperature and the abundance of dissolved alkaline earth and transition metals (e.g. Mg^2+^, Ca^2+^ and Fe^2+^, whose abundances are also likely limited by atmospheric pCO_2_)^72,73^.

The P content of the CC and OC appear to have remained relatively constant over recent Earth history^55^, which provides important model constraints. Again, this points to large mantle reservoirs dominating uncertainty in models and points to the importance of potential local enrichment being a phenomenon that cannot be addressed using global cycling models.

### Extraterrestrial input

Studies of the age and composition of impact craters of various Solar System bodies suggest that the inner planets received a large flux of extraterrestrial (ET) material as the Solar System formed ~ 4.5 Ga, which has since been declining exponentially over time^74^ approaching an asymptote^75^. On Earth this input was likely distributed among surface oceanic and emergent land reservoirs according to their surface area. We used estimates from^76^ to parameterize the flux and composition of ET P-delivering materials after the formation of the Earth-Moon system, which includes cometary, asteroidal, meteoritic, and interplanetary dust particle (IDP) inputs.

Some studies suggest that a putative Late Heavy Bombardment (LHB) period brought ~ 2 × 10^20^ to 5 × 10^22^ kg of ET material to the prebiotic Earth over a period of 10^7^ to 10^8^ years, possibly contributing a sizeable P mass to the surface of the primitive Earth^1,29,76^, though possibly much less. Such a “burst” of delivery is not modeled here, and in any event this flux^1^, estimated to have peaked ~ 2 × 10^8^ kg P year^−1^, gives a total LHB-delivered P mass of 2 × 10^15^ to 2 × 10^16^ kg P, which is small (on the order of 1% or less) compared to the initial surface reservoir P masses considered (see Table 1). It should be noted that some doubts have been raised regarding the timing and intensity of LHB flux^77^.

The time dependent flux of ET material was modeled using an exponential decay function (see^22^):

$$F(t)={F}_{0}+ ({F}_{1}-{F}_{0}) {exp}^{-t/\tau }$$

where ${F}_{0}$ and ${F}_{1}$ are the approximated minimum (modern) and maximum (initial) fluxes of P across all ET input types and $\tau$ is a t_1/2_ decay constant of 150 million years (Ma) derived from^78^. ${F}_{0}$ and ${F}_{1}$ were assigned values of 2.0 × ${10}^{5}$ kg P year^−1^ and 2.0 × ${10}^{8}$ kg P year^−1^ respectively based on^78^ (see Figure SI9), though these values can be altered in this workflow to explore other parameterizations.

### Ocean and atmosphere processes

Oceanic and atmospheric P cycling depends on the time-dependent content of P in each reservoir. There is a saturation concentration (e.g., the maximum solubility of phosphate in seawater or the maximal steady-state suspended atmospheric dust load) beyond which incoming P surpassing a specified value must rapidly move into another reservoir. These limits serve as rapid “switch points” between connected reservoirs, and they may thus be critical components of planetary P cycling. Finer variables in oceanic and atmospheric chemistry (e.g., deviations from modern oceanic pH, which might solubilize P, higher atmospheric density, which might allow suspended dust to persist longer, etc*.*) are not considered in this model, though such second-order dependencies can be modeled using its framework.

The P content of the modern atmosphere is mostly derived from aeolian CC erosion^61^, with a smaller contribution from oceanic sea spray (SS)^56,79^. This continuous P input into the atmosphere is principally removed by rain-out to the continental crust and oceans. We used estimates from^56^ of 2.8 × 10^7^ kg as the maximal steady-state atmospheric value for P, assuming rain-out rates balance a steady-state regardless of their source. The amount of P circulated through the atmosphere per year currently is ~ 200 times higher than the estimated current steady-state, ~ 4.6 × 10^9^ kg year^−1^, of which ~ 3.2 × 10^9^ kg year^−1^ is deposited to the continents and ~ 1.4 × 10^9^ kg year^−1^ to the oceans^56^.

Rain-out from the atmosphere is an important P input process for the modern oceans which drives numerous modern observable P abundance-dependent outcomes such as marine plankton blooms (e.g.^80^). Modern average CC aeolian erosion rates were estimated using the data in^56^ atmospheric P input rate of 4.0 × 10^9^ kg year^−1^ from the modern CC given its average P content and subaerial surface area. We used a surface-area averaged modern aeolian erosion rate of 27 kg km^−2^ year^−1^ P. This rate assumes a constant average aeolian erosion rate over an averaged CC lithological composition scaling with CC surface area to estimate the flux of P into the atmosphere.

The P flux to the atmosphere from the oceans due to SS is presently estimated^56^ to be ~ 3.3 × 10^8^ kg year^−1^, or ~ 1.0 kg year^−1^ km^−2^ based on the modern ocean surface area^57^ (~ 3.6 × 10^8^ km^2^). In this model, this flux was computed as the product of oceanic dissolved P in units of molarity and an assumed constant value of mobilized seawater as a function of ocean surface area in km^2^ year^−1^. Due to the inherent limitations caused by P insolubility in seawater, SS fluxes are generally globally trivial, though possibly temporally biologically significant.

The oceans connect many geochemical processes in this model. P concentrations in the modern oceans are largely controlled by biological uptake, while the P content of the prebiotic oceans were likely controlled by erosional input and phosphate solubility^71,88^. Presently, riverine input from CC erosion is the primary source of P to the oceans, and ~ 90% of this P mass accumulates on continental shelves^82,83,84^, supporting the notion of there being a tight coupling between CC P erosion, MS P content and accretion of P-rich MS to CC. In essence, this loop couples P from all sources that enter the MS reservoir back to the CC, minus what becomes subducted. This model discriminates between open ocean OC and continental margin OC via MS. OC to ocean fluxes are essentially zero since the oceans are generally constantly saturated with P.

This model assumes only ~ 10% of CC erosionally derived P deposits to OC, which attempts to capture the idea that riverine fluxes deposit erosionally delivered P into near-shore environments that cycle principally via CC accretion and subduction of MS.

Riverine fluxes presently input P to MS at a rate of ~ 4.1 × 10^12^ kg year^−1^^84,85^, or roughly 2.8 × 10^4^ kg P km^−2^ subaerial CC year^−1^. Earth’s river basins drain varied CC lithologies, and the bulk lithology of the CC has likely varied over time from mainly basaltic to significantly granitic (e.g.^86^). Nevertheless, despite apparent temporal increasing secular variation in CC igneous rock P content^69^, the bulk P content of average CC rocks appears to have only varied by a factor of 0.4 or so over the past ~ 3.5 Ga, and been relatively constant since ~ 2.5 Ga^87^.

Phosphate readily forms insoluble complexes with Ca^2+^ and Mg2+^88^, in particular the apatite series of minerals which are abundant accessory minerals on Earth^16^, as well as with Fe^2+^ (*e.g*., in the form of vivianite), which strongly affects the flux of P via Earth’s hydrologic cycle. The P content of the oceans is mainly determined by the solubility of apatite in its various forms (fluorapatite, chlorapatite, hydroxylapatite, etc*.*). P solubility in seawater depends on pH, temperature, and ocean chemical composition^81,88^. Wallmann^61^ suggested that the output flux of P from the oceans significantly exceeds the input flux in the modern oceans, meaning the oceans are currently undersaturated with respect to P. This may also be true for the ancient (3.2–1.9 Ga) oceans which contained P concentrations estimated to have been ~ 10–25% of modern day ocean concentrations^88^, though other studies suggest P was only limited after the Great Oxidation Event^89^. Delaney^90^ suggests a modern ocean dissolved P mass of 9.4 × 10^13^ kg, but we examined P concentrations based on the solubility of phosphate based on a wider range of values determined by solubility considerations (see below and Figure SI11).

The model presented can easily be adapted to explore the effect of varying P solubility over time due to changes in oceanic pH or the abundance of various atmospheric or dissolved species which may contribute to P solubility in the oceans.

The solubility of P in seawater was estimated using the equilibrium chemistry model of HSC Chemistry (Outokompu Research Oy, v 7.1), which has previously been used to explore ocean chemistry on Jupiter’s moon Europa^91^, and P chemistry in the solar nebula^92^. This code uses the GIBBS energy solver^93^ to determine equilibrium concentrations, based on an initial composition distributing into a set of allowed substances, arranged into solutional phases. The equilibrium distribution of 113 gases, solids (separated into 32 varied phases), and aqueous species was calculated, with a focus on soluble phosphate (as H_X_PO_4_^X-3^ (aq)). The system was modeled as follows: a 10 L quantity of air (consisting of CO_2_ and N_2_ in a 1:1 ratio, at 1 atm) equilibrated with 1 L of ocean water (55.6 mol) that was itself in contact with 1 mmol of both Ca_5_(PO_4_)_3_F and CaCO_3_. The ocean chemistry was set to a modern composition of Na, K, Mg, and Ca, added as their corresponding chloride salts, and the pH was maintained at 8 using a H_2_S/HS^-^ buffer to mimic modern-day ocean pH without being governed by other water–rock–atmosphere interactions. The temperature was set at 25 °C. It should be noted, changing the temperature from 0 to 100 °C only changes the solubility of P in the oceans by a factor of 20–50 or so depending on pH (see Figure SI11), and this effect is negligible compared to the relative sizes of the the oceanic and OC or CC reservoirs, which are ~ 10^5^ times larger. From these initial conditions, we explored the effect of changing air: water ratios, pressures, temperatures, pCO_2_, salt composition, and pH (without the sulfide buffer).

The compounds investigated included the gases CH_4_, CO, CO_2_, COS, H_2_, HCN, H_2_O, H_2_S, HSO, HSO_3_, H_2_SO, H_2_SO_4_, N_2_, NH_3_, NO, O_2_, OH, PO, PO_2_, and S. These were equilibrated with 1 L of water with the dissolved constituents H_2_O, CN^−^, CO, H^+^, CO_2_, CO_3_^2−^, Ca^2+^, CaOH^+^, Fe^3+^, Fe^2+^, FeO^+^, FeOH^2+^, FeOH^+^, FeSO4^+^, H_2_, HCN, H_2_CO_3_, HCO_3_^−^, H_3_PO_4_, HPO_4_^2−^, H_2_PO_4_^−^, H_2_S, HS^−^, HSO_4_^−^, Mg^2+^, MgOH^+^, HF, F^−^, Na^+^, Cl^−^, N_2_, NH_3_, NH_4_^+^, NO_2_^−^, NO_3_^−^, O_2_, OH^−^, PH_3_, PO_4_^3−^, S^2−^, and SO_4_^2−^. The solids investigated included CaCO_3_, CaHPO_4_ (monetite), CaHPO_4_ × 2H_2_O (brushite), Ca(OH)_2_, Ca_3_(PO_4_)_2_, Ca_5_(PO_4_)_3_F and Ca_5_(PO_4_)_3_OH (solid solution), CaSO_4_ × 2H_2_O and CaSO_4_ (solid solution), FeCO_3_, FeO, Fe_2_O_3_, Fe_3_O_4_, an iron oxyhydroxide solid solution system (consisting of Fe(OH)_2_, Fe(OH)_2_ (brucite), Fe(OH)_3_, Fe_2_O_3_ × H_2_O, FeO × OH, and FeO × OH (limonite)), FePO_4_ × 2H_2_O (strengite), and iron sulfate solid solution system (consisting of Fe_2_(SO_4_)_3_, FeSO_4_ × H_2_O, FeSO_4_ × 4H_2_O, and FeSO_4_ × 7H_2_O), a liquid acid solution consisting of H_3_PO_4_, H_3_PO_4_ × 0.5H_2_O, and H_2_SO_4_, MgCO_3_, MgFe_2_O_4_, MgO and Mg(OH)_2_ (solid solution), Mg_3_(PO_4_)_2_, MgSO_4_, Fe_3_(PO_4_)_2_ × 8H_2_O (vivianite), MgNH_4_PO_4_ × 6 H_2_O (struvite), MgHPO_4_ × 3H_2_O (newberyite), FeS and FeS_2_ (solid solution), Fe, S, Na_2_S (added with H_2_S to adjust pH to 8), NaHCO_3_ and Na_2_CO_3_ (solid solution), a solid solution of chloride salts (NaCl, MgCl_2_, FeCl_2_, CaCl_2_, NH_4_Cl, and KCl), CaF_2_, and NaOH (to adjust pH as needed). Thermodynamic data for these species was used in the HSC module and was supplemented by data for struvite, strengite, vivianite, and newberyite from Feng et al.^94^. Model results are shown in Figure SI11.

### Continental crust, oceanic crust and marine sediments

Modern MS has a mass of ~ 2.6 × 10^20^ kg, containing ~ 4 × 10^18^ kg P^51^. The main processes influencing the MS reservoir P content are inputs from dust deposition and CC erosional input (both of which depend on CC P abundance) and outputs from subduction and accretion fluxes. The subducted P flux (from the OC and a fraction of the MS deposited above it towards the UM, ${F}_{oc-um}$ and ${F}_{ms-um}$) and accretion P flux (from MS to the CC, ${F}_{ms-cc}$) were modeled using the following expressions from^22^:

$$F_{ms - um} = \left( {1 - \in } \right)\frac{{M_{sed} }}{\tau }$$

$$F_{sed - cc} = \in \frac{{M_{sed} }}{\tau }\quad F_{oc - um} = \frac{{M_{ocr} }}{\tau }$$

where $\epsilon$ is a constant accretion efficiency factor ranging between 0 and 1 which governs the fraction of the P subduction rate that gets accreted back to the CC, ${M}_{ms}$ is the mass of P in the MS, ${M}_{oc}$ is the mass of P in the OC, and $\tau$ is the modern subduction rate of complete turnover per 100 Ma, which is the OC recycling time^95^.

### Volcanism

Volcanism was modeled as a mechanism that distributes UM and LM material to crustal reservoirs in three ways: arc volcanism, hotspot volcanism, and mid-ocean ridge (MOR) volcanism (e.g. seafloor spreading). Presently, UM is estimated to add ~ 0.5 to 5 km^3^ year^−1^ via arc volcanism to the CC and ~ 20 km^3^ year^−1^ via MOR volcanism to the OC^96,97^. Modern hotspot (here presumed to be derived from LM) volcanism delivers ~ 2–2.5 km^3^ year^−1^ of LM material to both the OC and CC^97^, presumably split by the ratios of CC to OC surface area. Given the observed differences of mantle and volcanic lava P content^26^, we included a volcanism P enrichment factor (see Table 2), which attempts to reflect the way P becomes concentrated in various magmatic melt settings. This factor enriches various lavas as they erupt from different sources (UM and LM) and this variation impacts the final P-content values of surface reservoirs (CC, MS, OC). We have also explored the variation in the global reservoirs by using sensitivity analysis (see figures SI13, SI14, and SI15).

Given that the estimated redox state of Earth’s UM, which likely governs surface P redox state, appears to have been similar to that of the modern value over most of Earth history^98^, it can be assumed there has never been a significant volcanogenic flux of reduced P to the atmosphere. Presently, volcanically emitted P quickly condenses and precipitates locally, and P emitted from hydrothermal plumes quickly adheres to iron oxides in seawater and precipitates to the seafloor^58,61,99,100^. Besides volcanic dust, most transport processes quickly deposit their P contents to adjacent reservoirs.

P removal from ocean water to OC via hydrothermal circulation^100^ is included in this model. This is the only direct flux of P from the oceans into the OC in this model. This flux was modeled as exponentially decaying over geological time, in line with the estimated decay of oceanic hydrothermal circulation (e.g.^101^).

### Core, lower mantle and upper mantle

About 90% of Earth’s P is suggested to be stored in the core based on estimates of measured modern reservoir abundances and estimates of the P content of the material from which the Earth is thought to have formed^15^. Due to the siderophilicity of P, most models assume Earth’s P inventory rapidly accreted to its core and has not transported significantly upwards since^17^. Based on modern, albeit limited, understanding of deep Earth processes, we assume that ~ 90% of the global P budget is inaccessible to BSE reservoirs (e.g., the LM and UM, CC and OC, oceans and atmosphere, though alternative situations where 85, 95 and 97% of the same total planetary P inventory are sequestered in the core were also explored in Figures SI16 and 7). Deep Earth reservoir P exchange is mainly governed by mantle convection in this model. Laneuville et al.^22^ used mantle mixing rates of ~ 10^–9^ to 10^–8^ year^−1^ based on estimated modern-day convection speeds of 1–10 cm year^−1^ from^102,103^. These values were scaled to fit early Earth convection speeds, suggested to be more vigorous than at present^104^, to give a minimum mixing rate of 1 × 10^–8^ and a maximum rate of 3 × 10^–8^ year^−1^.

## Random seeding

We randomized initial reservoir seeding to allow the system to evolve independently from modern observed outcomes. Modeling started with a random distribution of an estimated initial BSE P mass of 8.4 × 10^20^ kg (assuming 90% of an initial P inventory is sequestered in the core, see Table 1) distributed over the seven considered BSE reservoirs, which were constrained with maximum limits for initial P composition as follows: atmosphere (2.8 × 10^7^ kg—the estimated steady state amount of dust and aerosol P in the modern atmosphere, see above), oceans (9.3 × 10^13^ kg P, dissolved in ~ 1.4 × 10^21^ L), CC and MS (~ 100 kg to avoid numerical errors while integrating). The randomly seeded models were then allowed to evolve over 9 × 10^5^ sequential 5000 year timesteps (for a total of 4.5 Ga).

## Supplementary Information

Supplementary Information.

## Data Availability

All unpublished data is available upon request.
