# Supplementary material for: A global network model of abiotic phosphorus cycling on Earth through time
Source: Sci Rep. 2022 Jun 7;12:9348. doi: 10.1038/s41598-022-12994-9 (PMC9174171; doi:10.1038/s41598-022-12994-9)
Supplement: Supplementary file 1 — Supplementary Information. [file 41598_2022_12994_MOESM1_ESM.pdf]

Supplementary Information for **A Global Network Model of Abiotic Phosphorus Cycling on Earth Through Time**

Marcos Jusino-Maldonado[1,2], Rafael Rianço-Silva[2,3], Javed Akhter Mondal[2,4], Matthew Pasek[5], Matthieu Laneuville[6], H. James Cleaves II[2,6,7]

Affiliations
1 Planetary Habitability Laboratory, University of Puerto Rico at Arecibo
2 Blue Marble Space Institute of Science
3 Departamento de Física, Faculdade de Ciências, Universidade de Lisboa, 1749-016 Lisboa, Portugal
4 Department of Geology, University of Calcutta, Kolkata-700019, India
5 University of South Florida
6 Earth-Life Science Institute, Tokyo Institute of Technology
7 Earth and Planets Laboratory, Carnegie Institution of Washington

The code for this model can be found on GitHub at https://github.com/Javi786/P-Cycle-Model.

Figure SI1. Model results assuming the core rapidly sequestered 85% of the estimated chondritic P input, but otherwise using the parameters presented in Table 2. Horizontal dotted lines represent best-guess modern reservoir values.

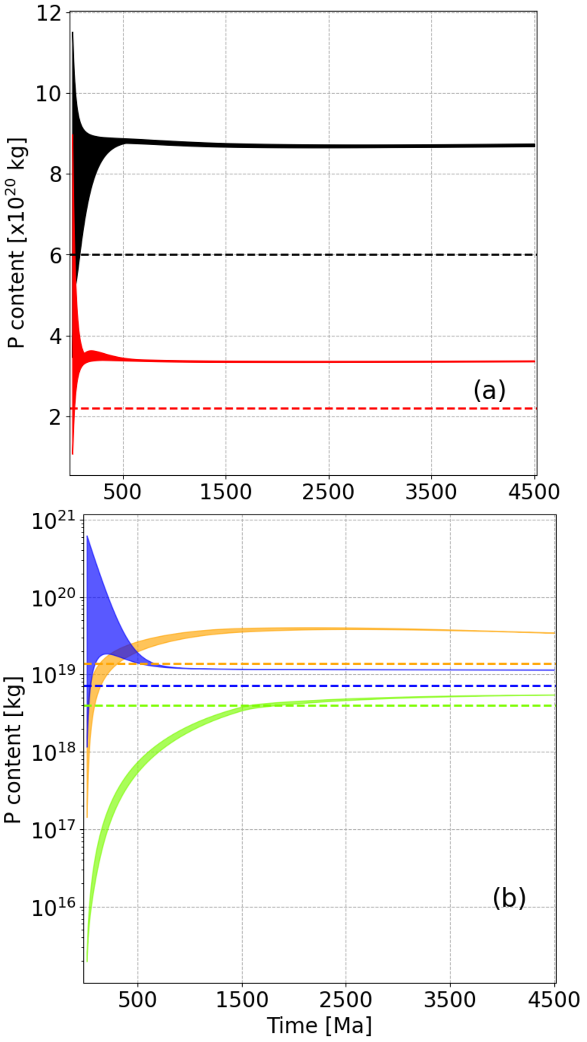

Figure SI2. Model results assuming the core rapidly sequestered 95% of the estimated chondritic P input, but otherwise using the parameters presented in Table 2. Horizontal dotted lines represent best-guess modern reservoir values.

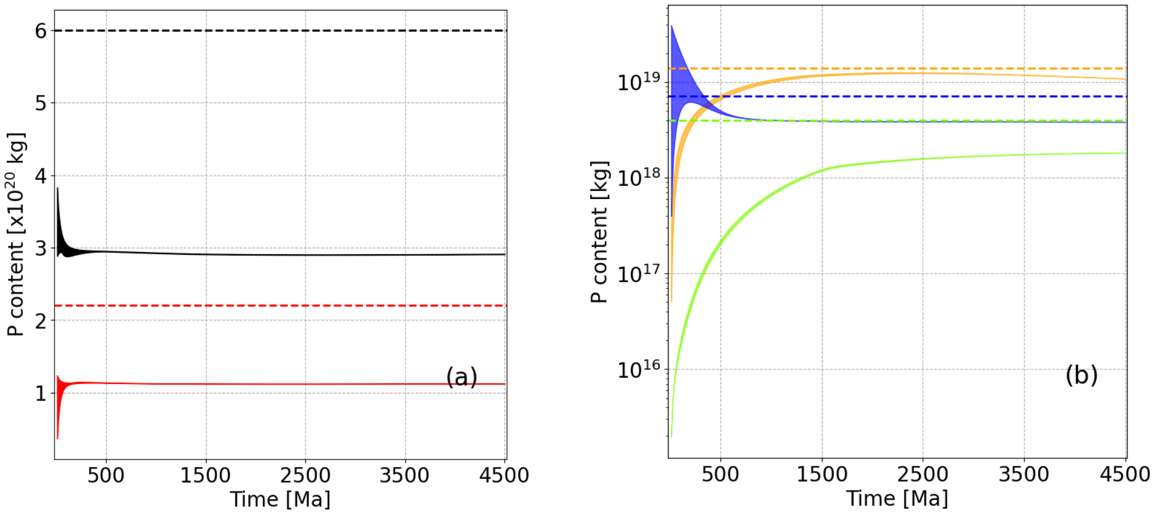

Figure SI3. Model results assuming the core rapidly sequestered 97% of the estimated chondritic P input, but otherwise using the parameters presented in Table 2. Horizontal dotted lines represent best-guess modern reservoir values.

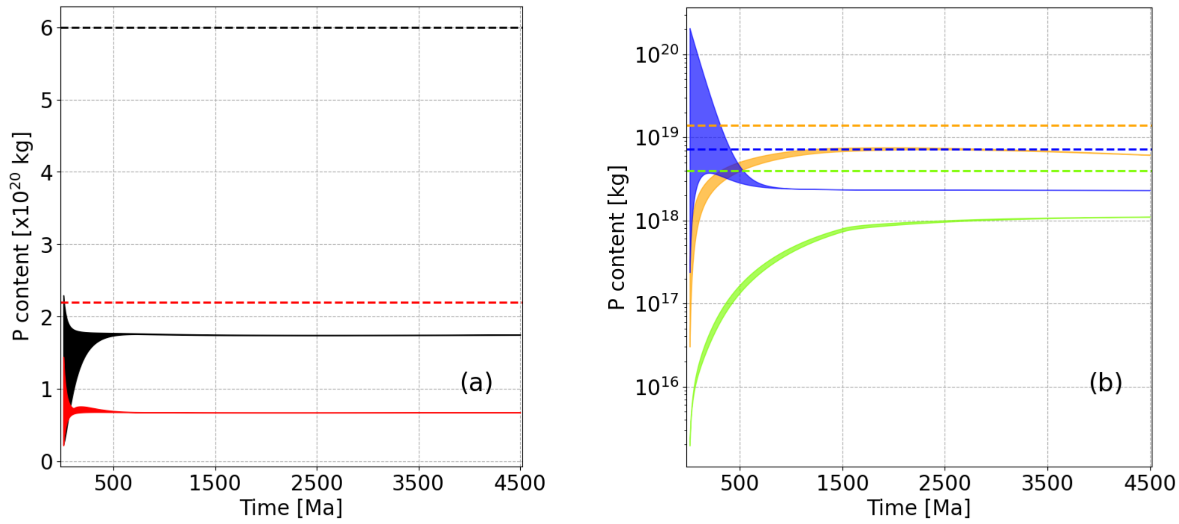

Figure SI4. Reservoir P mass as a function of maximum ocean P concentration for several subduction timescales according to the standard model presented using the parameters in Table 2 in the main text. Vertical dotted lines represent best-guess modern day values. (a) The impact of subduction timescales varied against maximum Oceanic P concentration on OC P content, (b) a) The impact of subduction timescales varied against maximum Oceanic P concentration on MS P content, (c) a) The impact of subduction timescales varied against maximum Oceanic P concentration on Oceanic P content.

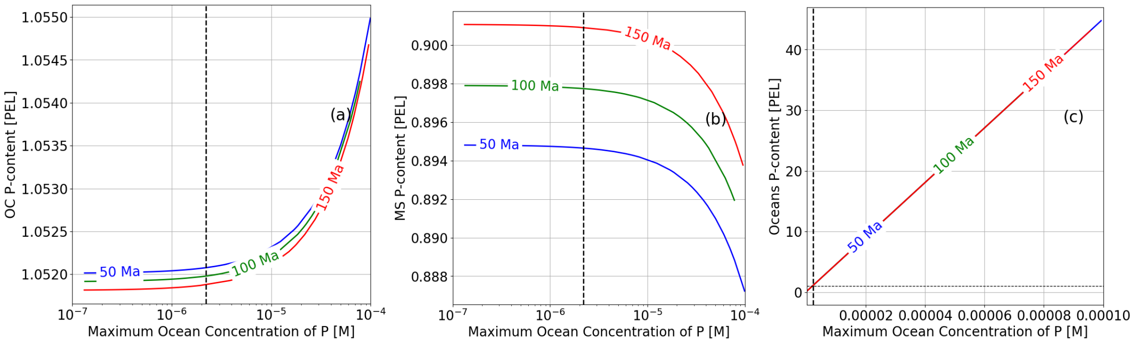

Figure SI5. Reservoir P mass as a function of erosion rate vs. other varied model parameters (see Table 2) according to the standard model presented in the main text. PEL = Present Earth Level (see Table 1). Vertical dotted lines represent best-guess modern day values. (a) The impact of erosion rate varied against accretion rate on UM P content, (b) The impact of erosion rate varied against accretion rate on CC P content, (c) The impact of erosion rate varied against OC turnover time on MS P content, (d) The impact of erosion rate varied against OC turnover time on UM P content, (e) The impact of erosion rate varied against OC turnover time on OC P content, (f) The impact of erosion rate varied against OC turnover time on MS P content, (g) The impact of erosion rate varied against a factor of increased volcanic activity on early Earth on UM P content, (h) The impact of erosion rate varied as a factor of increased volcanism on early Earth on OC P content, (i) The impact of erosion rate varied against a factor of increased volcanism on early Earth on CC P content.

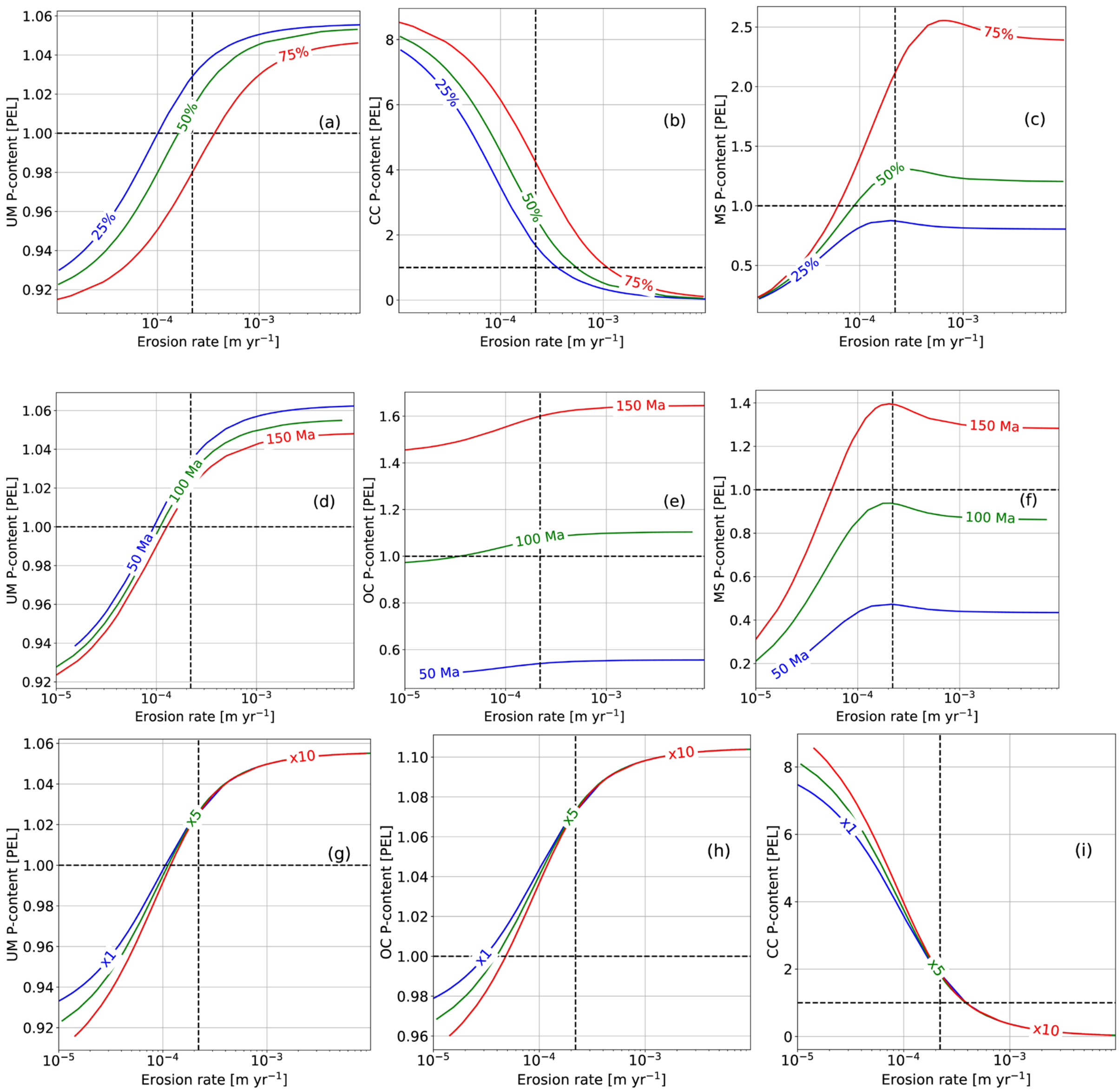

Figure SI6. Reservoir P mass for variable continental accretion efficiency relative to the standard model using the parameters in Table 2 in the main text as a function of CC accretion efficiency for varied mean OC subduction rates. Vertical dotted lines represent best-guess modern day values. (a) The effect of continental accretion efficiency vs. mean OC subduction rate for UM, (b) The effect of continental accretion efficiency vs. mean OC subduction rate for OC, (c) The effect of continental accretion efficiency vs. mean OC subduction rate for CC, (d) The effect of continental accretion efficiency vs. mean OC subduction rate for MS.

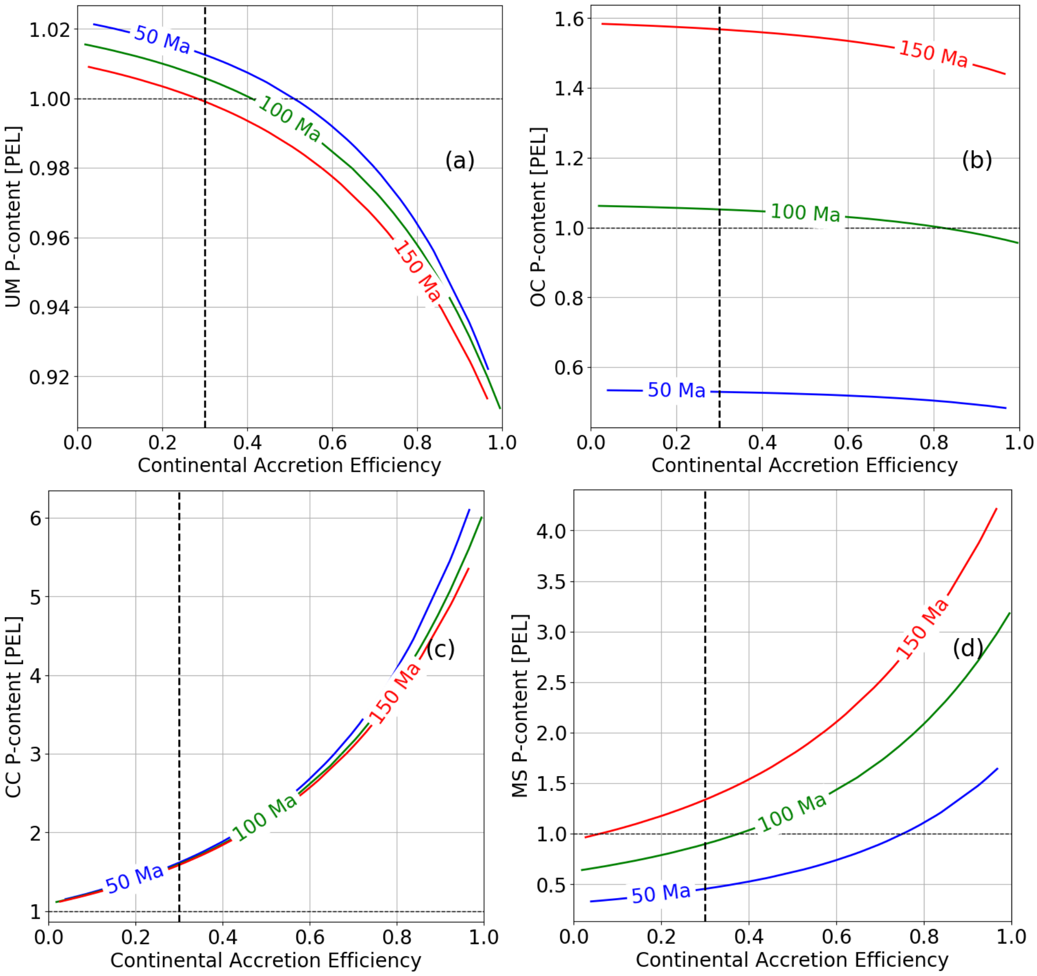

Figure SI7. Reservoir P mass as a function of mantle mixing rate, for variable initial volcanism rates relative to the standard model were computed using the parameters in Table 2 in the main text. Vertical dotted lines represent best-guess modern day values. (a) The effect of mantle mixing rate vs. variable initial volcanism rate for LM, (b) The effect of mantle mixing rate vs. variable initial volcanism rate for UM, (c), The effect of mantle mixing rate vs. variable initial volcanism rate for OC, (d), The effect of mantle mixing rate vs. variable initial volcanism rate for CC, (e), The effect of mantle mixing rate vs. variable initial volcanism rate for MS.

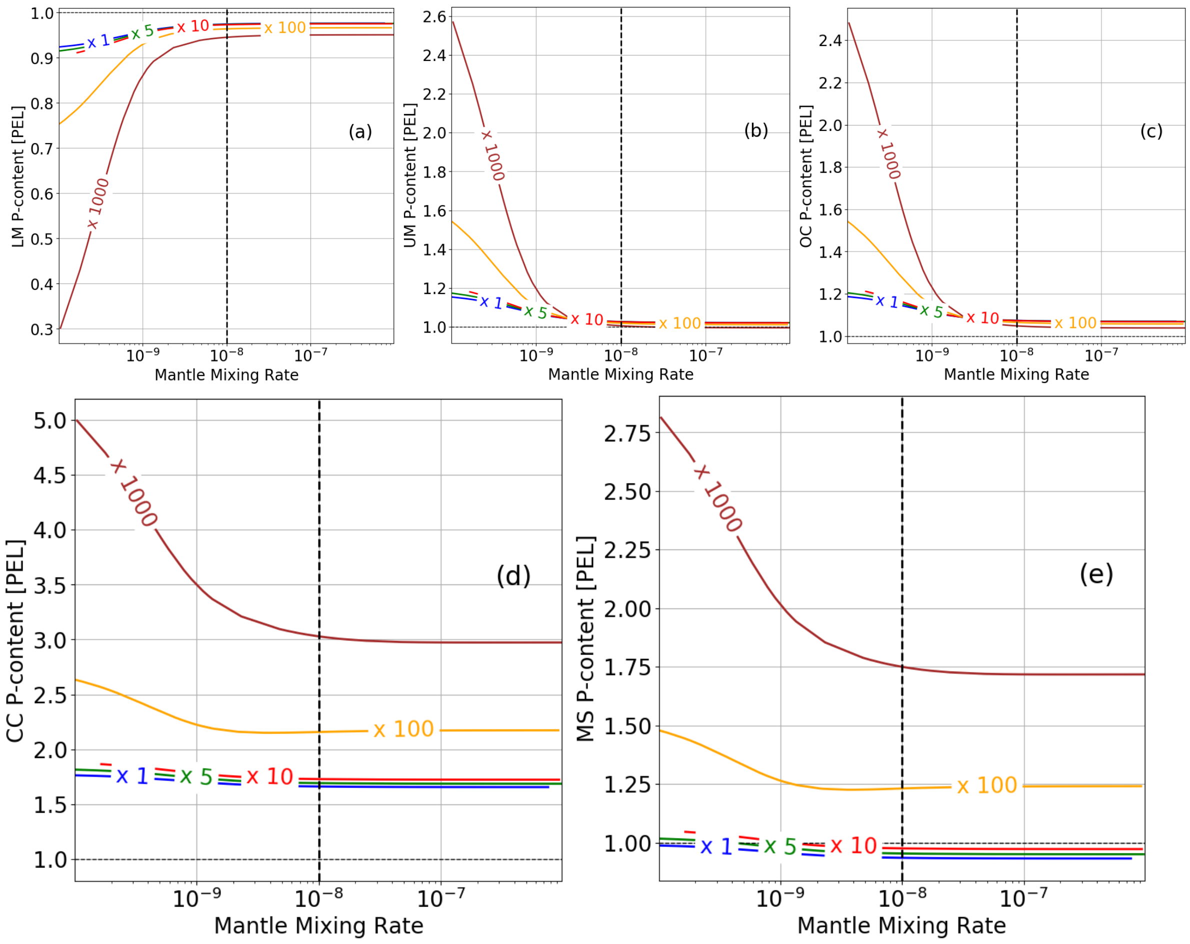

Figure SI8. Plots showing P fluxes through various flux channels as a function of time in the standard model. Note that in (a) the modern estimated arc and hotspot volcanism fluxes are superposed, and in (b) the modern estimated subduction and erosion fluxes are superposed.

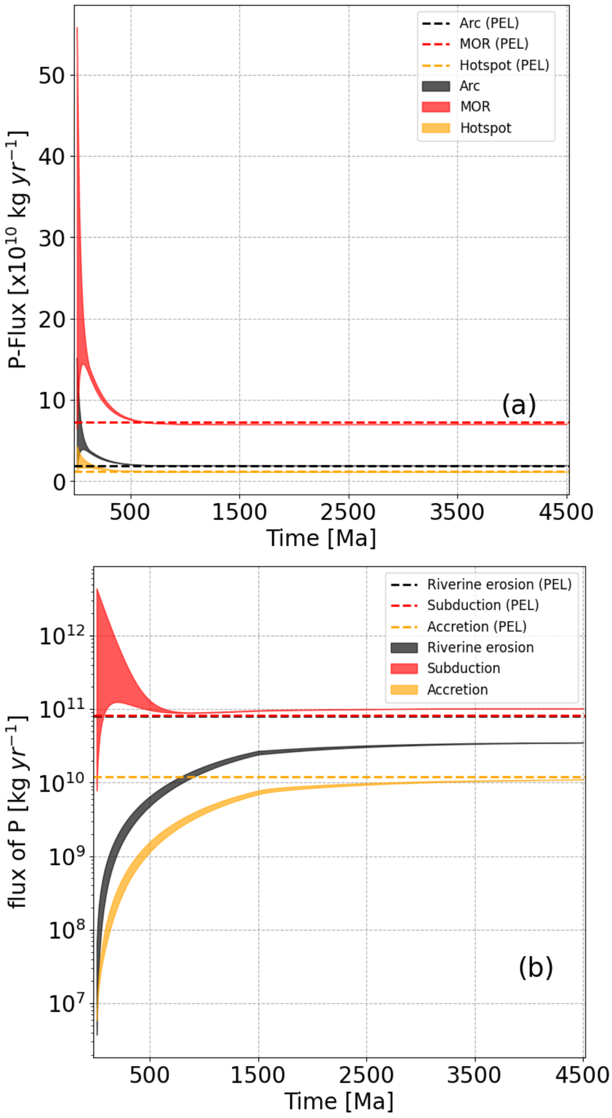

Figure SI9. The ET P flux parameterized according to Chyba and Sagan (1992) used in this model. The horizontal dotted line represents the estimated modern flux.

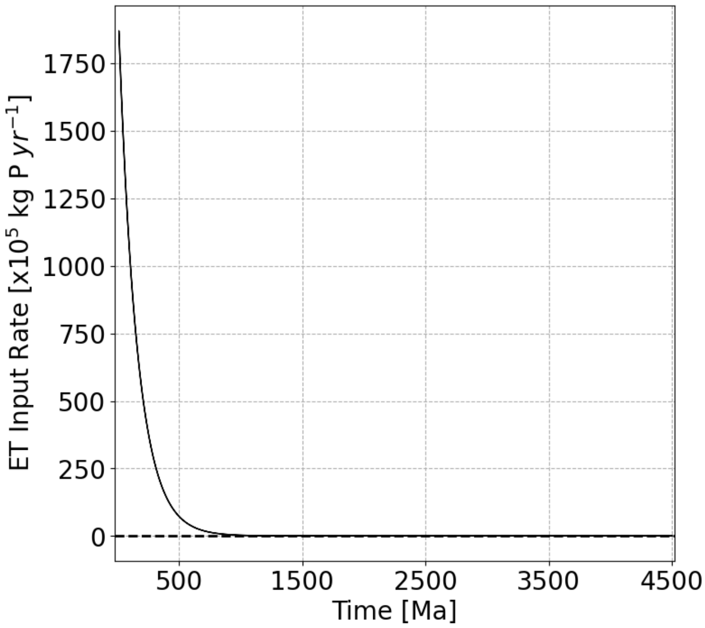

Figure SI10. Model results assuming low-erosional rate ($5x10^{-6}$) in the 90% core value of the estimated chondritic P input. The horizontal dotted lines represent the best-guess modern day values.

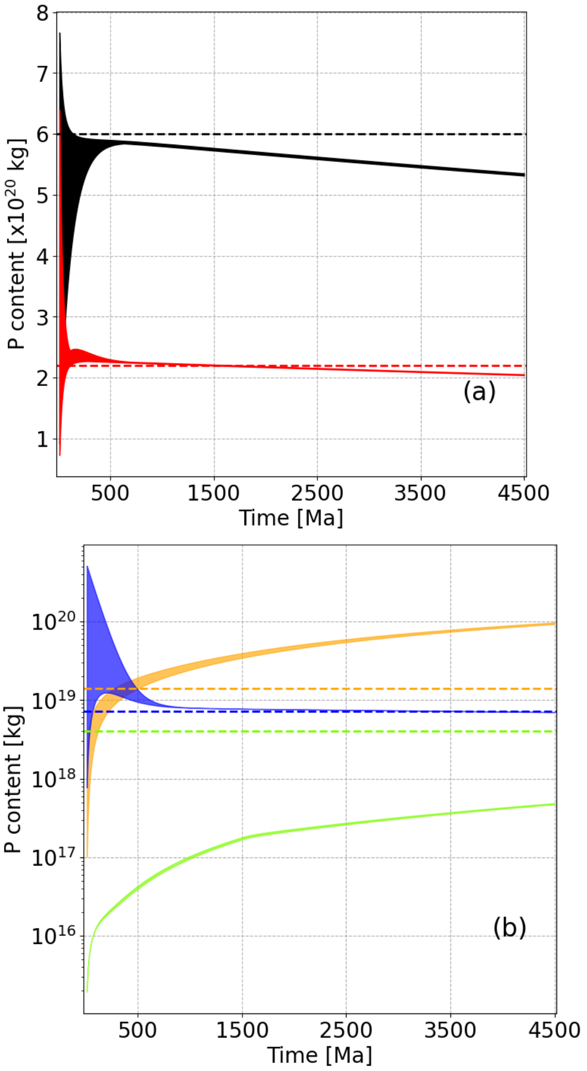

Figure SI11. The solubility of phosphate modeled by HSC Chemistry showing that P is in general only slightly affected by aqueous conditions for a range of plausible early Earth environmental variables. The largest change in solubility under most conditions is due to temperature, which increases apatite solubility by a factor of roughly 30 from 0°C to 100°C. Varying ratios of inorganic salts, changing the composition of the atmosphere, and modifying the gas:liquid ratio generally has little effect. The largest effect from aqueous chemistry, shown only at 25°C, is varying pH: decreasing the pH from 8 to 2 greatly enhances apatite solubility, to the point where apatite is completely dissolved at pH 2. Unless otherwise stated, the atmospheric composition was set to 1:1 CO_2:N_2, the gas:liquid ratio (volume:volume) was 10:1, the temperature ranged from 0-100°C, the aqueous phase (1 L) was modeled in equilibrium with 1 mmole of apatite (0.5 g) and 1 mmole of $CaCO_3$ (0.1 g). Water composition was set to pH 8, with 600 mM Na^+, 40 mM Mg^{2+}, and 10 mM Ca^{2+} and K^+, with no Fe^{2+} or NH_4^+.

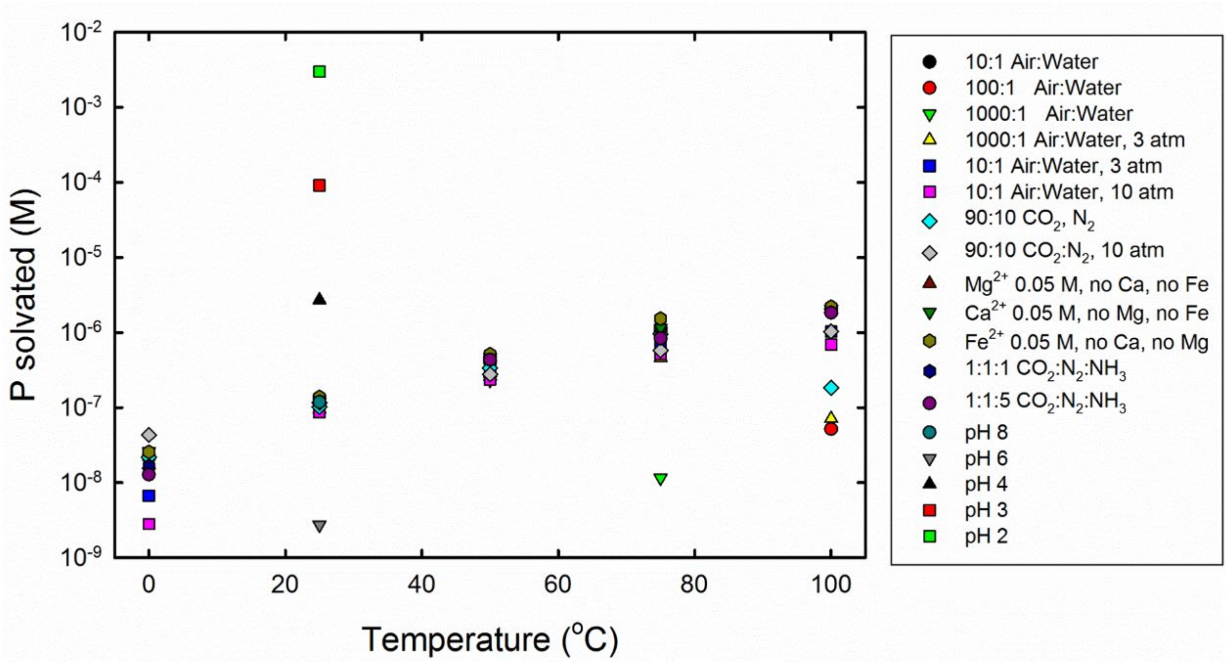

Figure SI12. Model results assuming an alternative scenario in which CC growth rate decays exponentially in the 90% core value of the estimated chondritic P input. The horizontal dotted lines represent estimated modern day values.

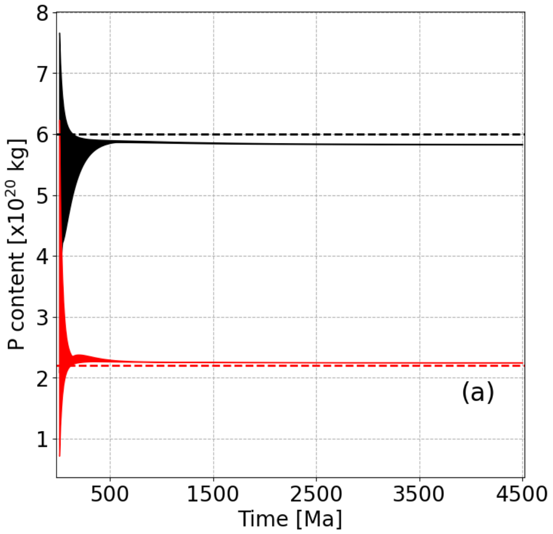

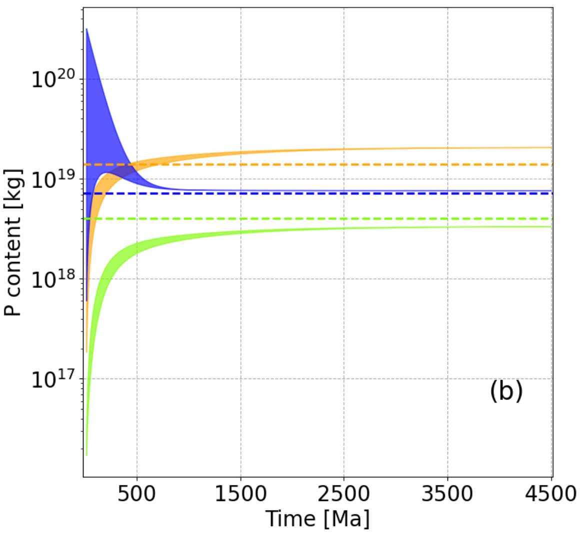

Figure SI13. Reservoir P mass as a function of erosion rate vs. MOR volcanism P enrichment factor. PEL = Present Earth Level (see Table 1). Horizontal dotted lines represent estimated modern reservoir values; vertical dotted lines represent estimated modern P fluxes. (a) Impact of erosion rate varied against the volcanism P enrichment factor on CC P content, (b) Impact of erosion rate varied against the volcanism P enrichment factor on OC P content, (c) Impact of erosion rate varied against the volcanism P enrichment factor on MS P content, (d) Impact of erosion rate varied against the volcanism P enrichment factor on UM P content.

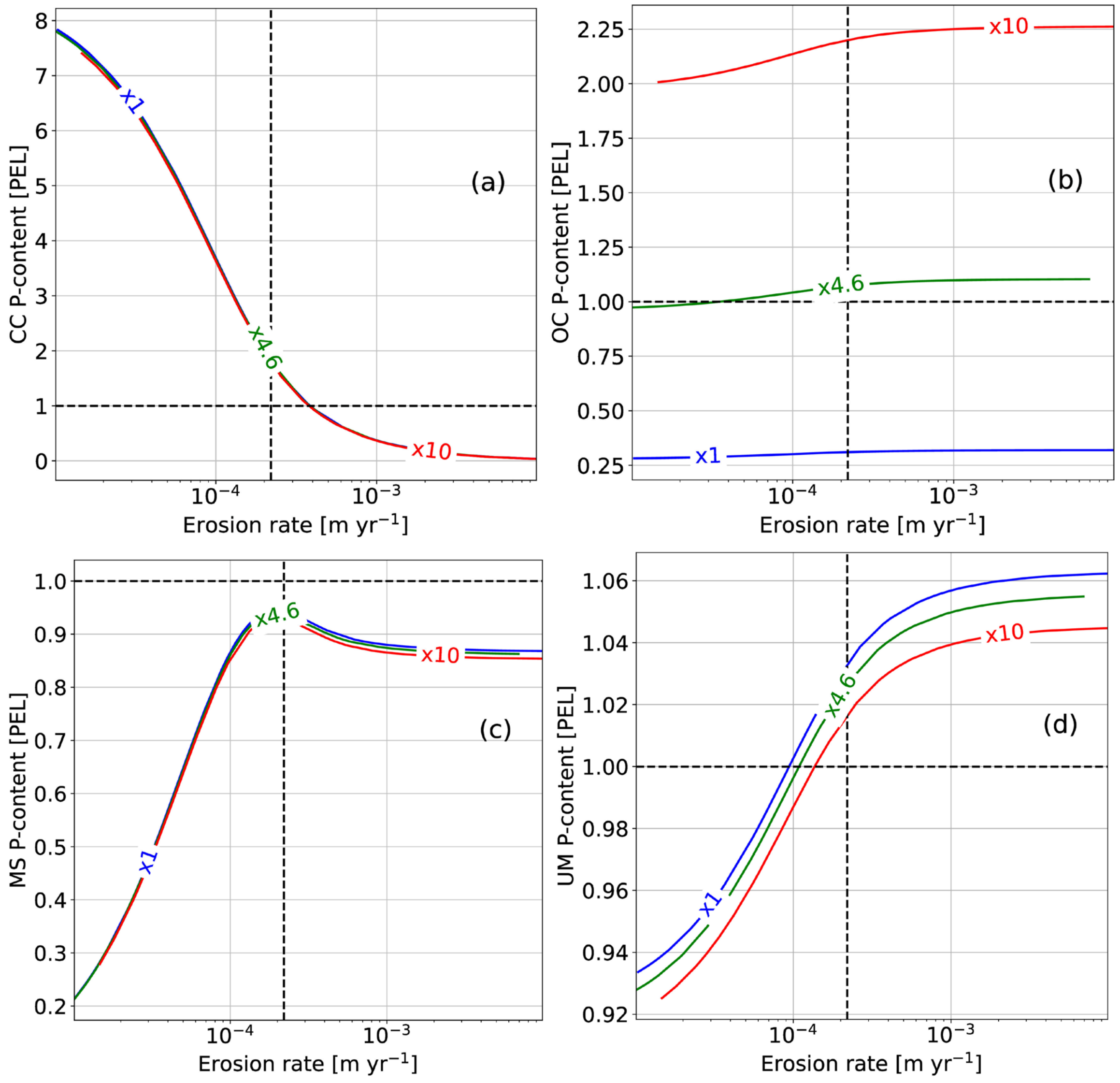

Figure SI14. Reservoir P mass as a function of MOR volcanism P enrichment factor vs Initial Volcanism factors. PEL = Present Earth Level (see Table 1). Horizontal dotted lines represent estimated values of modern reservoirs; vertical dotted lines represent estimated modern P fluxes. (a) The impact of P-enrichment factors against the initial volcanism factor on UM P content, (b) The impact of P-enrichment factors against the Initial volcanism factor on OC P content, (c) The impact of P-enrichment factors against the initial volcanism factor on CC P content, (d) The impact of P-enrichment factors against the initial volcanism factor on MS P content.

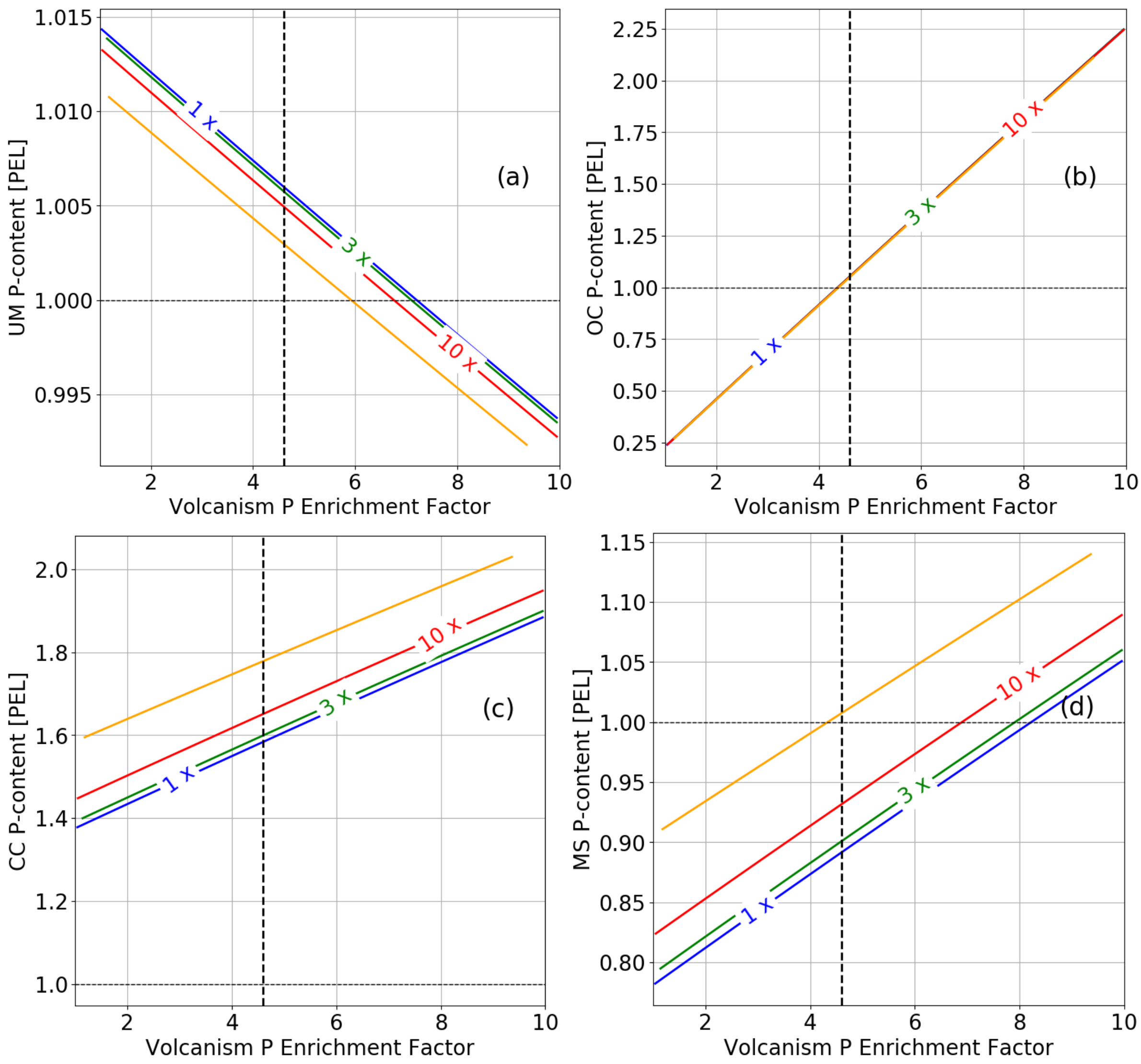

Figure SI15. Reservoir P mass as a function of the MOR volcanism P enrichment factor vs OC subduction rate. PEL = Present Earth Level (see Table 1). Horizontal dotted lines represent estimated modern reservoir values; vertical dotted lines represent estimated modern P fluxes. (a) The impact of volcanism P enrichment factor vs. the OC subduction rate time on UM P content, (b) The impact of volcanism P enrichment factor against the OC subduction rate time on OC P content, (c) The impact of volcanism P enrichment factors against the OC Subduction Rate Timescales on MS P content.

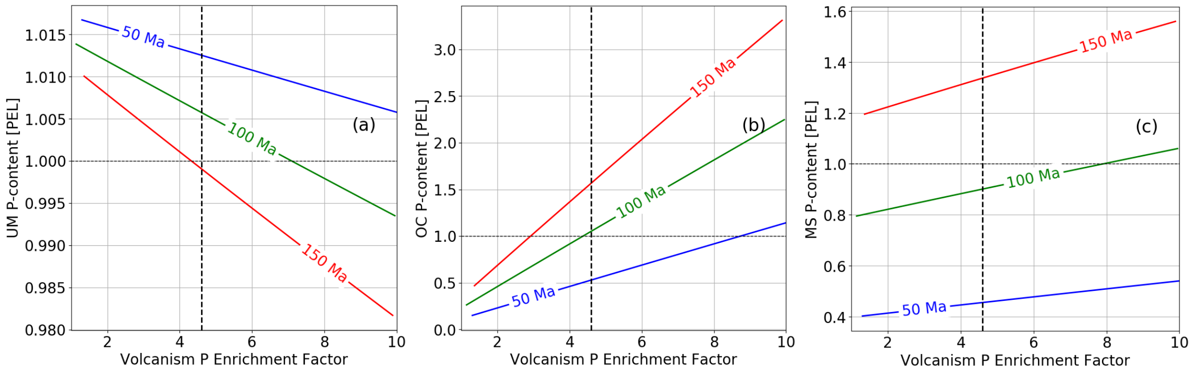

References

Chyba, C. & Sagan, C. Endogenous production, exogenous delivery and impact-shock synthesis of organic molecules: an inventory for the origins of life. *Nature*, **355**, 125-132 (1992).
